# Long-Term Survival of Older Patients Hospitalized for COVID-19. Do Clinical Characteristics upon Admission Matter?

**DOI:** 10.3390/ijerph182010671

**Published:** 2021-10-12

**Authors:** Michał Chojnicki, Agnieszka Neumann-Podczaska, Mikołaj Seostianin, Zofia Tomczak, Hamza Tariq, Jerzy Chudek, Sławomir Tobis, Iwona Mozer-Lisewska, Aleksandra Suwalska, Andrzej Tykarski, Piotr Merks, Sylwia Kropińska, Małgorzata Sobieszczańska, Frank Romanelli, Katarzyna Wieczorowska-Tobis

**Affiliations:** 1 Department of Immunobiology, Poznan University of Medical Sciences, 60-806 Poznan, Poland; mchojnicki@gmail.com; 2 Department of Infectious Diseases, Jozef Strus Hospital, 61-285 Poznan, Poland; iwonalisewska@poczta.onet.pl; 3 Geriatric Unit, Department of Palliative Medicine, Poznan University of Medical Sciences, 61-245 Poznan, Poland; ar-n@wp.pl (A.N.-P.); z.t.tomczak2@gmail.com (Z.T.); hamza_tariq@hotmail.co.uk (H.T.); skropins@ump.edu.pl (S.K.); kwt@tobis.pl (K.W.-T.); 4 Department of Internal Medicine and Oncological Chemotherapy, Medical University of Silesia in Katowice, 40-027 Katowice, Poland; chj@poczta.fm; 5 Department of Occupational Therapy, Poznan University of Medical Sciences, 60-781 Poznan, Poland; stobis@ump.edu.pl; 6 Department of Infectious Diseases, Hepatology and Acquired Immunodeficiencies, Karol Marcinkowski University of Medical Sciences, 61-285 Poznan, Poland; 7 Department of Mental Health, Chair of Psychiatry Poznan University of Medical Sciences, 60-572 Poznan, Poland; asuwalska@gmail.com; 8 Department of Hypertensiology, Angiology and Internal Medicine, Poznan University of Medical Sciences, 61-848 Poznan, Poland; tykarski@o2.pl; 9 Collegium Medicum, Faculty of Medicine, Cardinal Stefan Wyszyński University, 01-938 Warsaw, Poland; p.merks@uksw.edu.pl; 10 Department and Clinic of Geriatrics, Wroclaw Medical University, 50-369 Wroclaw, Poland; malgorzata.sobieszczanska@umed.wroc.pl; 11 Department of Pharmacy Practice and Science, University of Kentucky College of Pharmacy, Lexington, KY 40536, USA; frank.romanelli@uky.edu

**Keywords:** 180-day survival, COVID-19, older adults, functional impairment, prognosis

## Abstract

Older adults are particularly susceptible to COVID-19 in terms of both disease severity and risk of death. To compare clinical differences between older COVID-19 hospitalized survivors and non-survivors, we investigated variables influencing mortality in all older adults with COVID-19 hospitalized in Poznań, Poland, through the end of June 2020 (*n* = 322). In-hospital, post-discharge, and overall 180-day mortality were analyzed. Functional capacity prior to COVID-19 diagnosis was also documented. The mean age of subjects was 77.5 ± 10.0 years; among them, 191 were females. Ninety-five (29.5%) died during their hospitalization and an additional 30 (9.3%) during the post-discharge period (up to 180 days from the hospital admission). In our study, male sex, severe cognitive impairment, underlying heart disease, anemia, and elevated plasma levels of IL-6 were independently associated with greater mortality during hospitalization. During the overall 180-day observation period (from the hospital admission), similar characteristics, excluding male sex and additionally functional impairment, were associated with increased mortality. During the post-discharge period, severe functional impairment remained the only determinant. Therefore, functional capacity prior to diagnosis should be considered when formulating comprehensive prognoses as well as care plans for older patients infected with SARS-CoV-2.

## 1. Introduction

During the early stages of the COVID-19 pandemic, it became apparent that poorer clinical outcomes were seen in older patients with SARS-CoV-2 infection. A report issued by the Centers for Disease Control and Prevention (CDC) indicated that older adults constituted around 53% of intensive care unit (ICU) admissions and 80% of fatalities due to COVID-19 [1]. Their vulnerability to infection was further demonstrated in a study of 281,461 individuals performed by Li et al., where older patients were found to be more prone to a severe course of disease and death; non-survivors were approximately 20 years older than survivors (68.9 vs. 50.7 years) [2].

Although advanced age is a well-established independent risk factor for COVID-19, susceptibility to a SARS-CoV-2 infection and its consequences has also been associated with other clinical characteristics, such as pre-existing comorbidities, which is notable as the prevalence of comorbidities increases with age [3,4]. In a study by Jain and Yuan, chronic obstructive pulmonary disease (COPD) and cardiovascular disease, including hypertension, were predictive of both a severe course of COVID-19 and of ICU admission [5]. Moreover, cardiovascular and pulmonary disease, as well as diabetes mellitus, were found to increase fatality rates, independent of advanced age [6]. Collectively, this indicates that older patients are more likely to progress to severe COVID-19 with an associated higher risk of death, especially if their condition is worsened by underlying chronic conditions [7].

Apart from age and comorbidities, other clinical and laboratory characteristics have been found to increase the risk of an unfavorable COVID-19 course and the rate of mortality. A meta-analysis performed by Zheng et al., involving a group of 3027 patients, identified several laboratory markers as being potentially predictive of a more complicated course of COVID-19 infection [8]. A systematic review by Izcovich et al. identified smoking, increased body mass index (BMI), cerebrovascular disease, chronic kidney disease, cardiac arrhythmia, cancer, dementia, and low platelet counts as prognostic factors for COVID-19 mortality [9].

In February 2021, the UK Office for National Statistics identified disability status as an important feature of risk of death among patients infected with COVID-19 [10]. Disabled people made up nearly six out of every ten deaths related to COVID-19 (30,296 of 50,888 deaths—59.5%) [10]. Additionally, both a study performed by Mendes et al., involving 364 older Caucasian patients with COVID-19, and by Neumann-Podczaska et al., which included the first 50 older patients hospitalized for COVID-19 in Poznań, revealed that functional status was among the most important factors influencing mortality [11,12].

Older adults are particularly susceptible to COVID-19 in terms of both disease severity and risk of death. Therefore, we not only aimed to investigate the clinical characteristics of older subjects affected by the virus, but also to acknowledge how they are associated with long-term survival. Patient characteristics assessed upon hospital admission and their impact on 180-day survival could provide valuable insights for providers to consider when stratifying the risk of death due to COVID-19 both during the patient’s hospital stay and after discharge. Understanding the role(s) of these characteristics could also better inform the level of follow-up care and monitoring provided for these patients upon discharge.

## 2. Materials and Methods

### 2.1. Study Design

This was a single-center prospective register observational study, including all consecutive older patients (≥60 years of age) hospitalized in the Jozef Strus Hospital located in Poznan, Poland for COVID-19, between 12 March 2020 (the day when the first COVID-19 patient was admitted to the hospital) and 30 June 2020. Following epidemiological regulations, 19 hospitals in Poland were converted into COVID-19-dedicated units. These hospitals served COVID-19 patients solely, as did the Jozef Strus Hospital.

The study was approved by the Bioethics Committee of the Poznan University of Medical Sciences (KB 380/20 and KB 53/21). As it was a register study, no written consent from participants was required.

### 2.2. Patients

We analyzed the data of all older (≥60 years of age) patients who were consecutively admitted and hospitalized for over 48 hours in the aforementioned period. Altogether, 322 subjects were included (191 females and 131 males). Considering this limited population, stratification by age could not be achieved. The cut-off value of 60 years for older adults was based on the definition of an older adult as defined by the World Health Organization (WHO) [13].

All hospitalized subjects tested positive for SARS-CoV-2 by RT-PCR (in biological material of a nasopharyngeal swab, ORF1 ab and N genes were tested with declared sensitivity above ten viral copies as a measure to standardize testing). The Vitassay^®^ assay was used at Cobas^®^ 2480 (Roche) system, and Geneproof^®^ was used to isolate RNA.

Hospitalized subjects with severe COVID-19 symptoms were both community-dwelling and previously institutionalized, including inhabitants of nursing homes, retirement facilities, and hospice care centers, as well as patients from non-COVID hospitals. At the time, no official criteria for hospitalization of patients with COVID-19 were available. Decisions to admit were made upon triage performed by experienced internal medicine specialists based on clinical presentation as well as laboratory and imaging results.

### 2.3. Data Collection

For each patient, demographic information (age, sex, and place of residence: home/institution), clinical status and laboratory results on admission, and hospitalization outcomes were obtained. We also verified whether patients were previously hospitalized elsewhere for other reasons before their COVID-19 diagnosis.

Clinical manifestations of disease upon admission consisted of fever, cough, dyspnea, and myalgias. Information related to dysosmia and dysgeusia were also collected. Fever was defined as a body temperature ≥38.0 °C, while subfebrile temperature was understood to be between 37.1–37.9 °C.

Any cognitive disturbances were assessed by a standardized provider approach on admission. Observed cognitive disturbances were assigned to the following categories: normal (no signs of cognitive impairment; unaffected cognition), mild (partially preserved cognitive functions; including, but not limited to, clouding of consciousness, mild cognitive impairment, early/middle stage dementia, or delirium), or severe (late-stage dementia or unconsciousness). Cognitive disturbances were assessed by the attending physician and noted in the patients’ medical history. Data related to observed cognitive disturbances were extracted from the medical history by two geriatric professionals who are members of European Academy for Medicine of Ageing (EAMA). Thereafter, categories of cognitive disturbances were assigned according to the DSM-5 classification of neurocognitive disorders [14].

Past medical history and comorbidities were retrieved from medical records upon admission and categorized into cardiovascular diseases (hypertension, congestive heart failure, ischemic heart disease, arrhythmias, and valvular diseases), stroke, respiratory diseases (chronic obstructive pulmonary disease or asthma), diabetes, hepatic and/or renal dysfunction, and cancer (treated within the last five years). Only comorbidities and complications found in patients upon hospital admission were taken into consideration during the analysis.

Blood samples obtained from participants were examined as part of the laboratory work-up at admission. The findings included in the analysis were: white blood cells (WBC), lymphocytes (L) and lymphocyte percentages (%), neutrophils (Ne), as well as hemoglobin (HGB), platelet count (PLT), and lactate dehydrogenase (LDH). Parameters indicative of inflammation, such as C-reactive protein (CRP), procalcitonin (PCT), and interleukin 6 (IL-6), were also analyzed. Anemia was defined as a hemoglobin level below the normal range and corrected by gender (<12 g/dL for females and <14 g/dL for males according to the cut-off values in the hospital laboratory). Only measurements of hemoglobin taken upon hospital admission were considered during the analysis. In addition, kidney function was determined. Diminished renal function was scored if the estimated glomerular filtration rate (eGFR) calculated with the use of MDRD equation fell below 60 mL/min/1.73 m^2^. Severe renal disease was defined as eGFR < 30 mL/min/1.73 m^2^.

Two imaging modalities, including chest X-ray and CT (computer tomography) scans, were used to detect pathological lung changes. All radiologic analyses were interpreted by registered radiologists. Radiographic imaging performed more than 24 hours after hospital admission was not included in the analysis. Similarly, descriptive results of radiographic imaging performed in the hospital from which a patient was referred were not considered.

Based on medical history at admission, functional capacity prior to hospitalization was categorized and assessed in each patient. The assignment of functional capacity was performed by two experienced geriatric professionals (the members of EAMA). Functional status categories were based on activities of daily living (ADL) and instrumental activities of daily living (IADL) scores by Katz and Lawton [15]. The following functional status categories were established: no functional impairment (patient presented no dependence in basic and instrumental activities of daily living), mild functional impairment (patient required partial assistance in performing basic living activities or remained unable to perform instrumental activities of daily living), and severe functional impairment (patient required assistance in each basic activity of daily living). In cases of disagreement or uncertainty, an infectious disease specialist subsequently contacted the patient or the family/caregiver to clarify. As many as 139 differing opinions were identified while assessing patients’ functional status. In total, 43 phone calls to community dwelling patients/families were made. Nursing home staff were reached 96 times to clarify patient status. Eventually, a consensus was reached concerning the functional capacity of all patients, and each individual was assigned a functional status category.

Based on the dates of admission and discharge (or death), the length of hospitalization was calculated. Data related to patients who died during hospitalization were collected in the mortality record, and the rate of in-hospital death was calculated. For discharged patients, 180-day mortality (calculated from the day of admission) was assessed using the hospital system, in which patients’ death was immediately visible when reported by the national social security system. If a patient’s status remained unclear, the registration office was consulted since hospital systems in Poland do not track the health status (including death) of all discharged patients. Eventually, the dispositions of all hospitalized patients were resolved.

Data imputation was not performed. The data entered into our database was cross-checked and controlled for logical integrity.

### 2.4. Statistical Analysis

The primary tool for statistical analysis was the STATISTICA 13.0 package (by Tibco Software Inc., Palo Alto, CA, USA).

The Shapiro–Wilk test was used to assess the pattern of distribution of the variables. Continuous data were displayed as means ± standard deviation (SD). Due to the lack of normality of some of the variables, the median and range were also provided. No data imputation was performed. The percentage of patients with analyzed parameters were also calculated, assuming that 100% represents all patients in whom the specific parameters were assessed. The Mann–Whitney U test and t-test were utilized to compare continuous variables. Categorical variables were presented as numbers and percentages (%) and compared with the χ2 test with the Yates correction applied.

Two types of analyses were performed. The first compared patients who died during hospitalization (in-hospital non-survivals) and those who were discharged. The other compared patients who died in the post-discharge period up to 180 days after hospital admission (post-discharge non-survivors) with those who survived 180 analyzed days (survivors). Thirty-four patients did not have a documented IL-6 level. Therefore, the models for in-hospital and overall 180 day mortality included 288 patients. The post-hospital mortality model included all 322 subjects.

Factors affecting in-hospital mortality, post-discharge mortality, and overall 180-day mortality were evaluated using both univariable and multivariate Cox proportional hazard. An age cut-off of 75 years and older was set based on the demographics of older adults in European populations [13]. Variables found to be significantly different among studied groups were included in the model and presented as hazard ratios (HRs) with 95% confidence intervals (CI). All continuous variables were dichotomized for the purpose of logistic regression analysis. For inflammatory markers, cut-off values predicting in-hospital death were calculated with receiver operating characteristic (ROC) curves. Accordingly, cut-off values were determined along with assigned sensitivity and specificity. Additionally, overall 180-day mortality, including both in-hospital and post-discharge fatal outcome, were analyzed using Kaplan–Meier curves. A *p*-value below 0.05 was considered statistically significant.

## 3. Results

### 3.1. Baseline Characteristics

Patient characteristics are presented in Table 1. The study group included 322 patients (191 females, 61.8%) with a mean age of 77.5 ± 10.0 (median 77; range 60–101). Most patients were transferred from nursing homes (*n* = 163, 50.6%) or non-COVID-19 hospitals (*n* = 85, 26.4%); only 74 (23.0%) were admitted from home.

Chief complaints present during initial examination were subfebrile temperature (*n* = 94, 32.8%), cough (*n* = 66, 26.4%), and dyspnea (*n* = 54; 20.5%). Dysosmia and taste impairment (dysgeusia) were reported very rarely (*n* = 2 (1.3%) and *n* = 5 (3.3%), respectively). Among the subjects, 147 patients had cognitive impairment (45.7%); of these, 47 (14.7%) were diagnosed as having delirium on admission, and 30 were unconscious (9.4%). As far as dependence is concerned, 97 (30.4%) were partially, and 112 (35.1%) were totally, dependent.

History of cardiovascular diseases was reported in 265 (82.3%) subjects, including 199 (62.1%) with hypertension and 189 (59.3%) with other cardiovascular diseases. Ninety-nine patients were diagnosed with diabetes (31.0%). Other comorbidities were less common (Table 1). The mean number of analyzed comorbidities per patient was 3.2 ± 2.0 (median 3; range 0–11), and only 21 (6.5%) had no concomitant diseases.

Laboratory parameters are presented in Table 2. Hemoglobin levels were below reference values in 180 (57.3%) subjects. Inflammatory markers were above reference values in several patients (CRP; *n* = 275 (88.1% of patients in whom it was assessed), procalcitonin; *n* = 256 (90.5%), and IL-6: *n* = 237 (82.0%)). An eGFR below 60 mL/min/1.73 m^2^ was found in 106 subjects (33.8%), including 22 (7.0%) in whom the values were below 30 mL/min/1.73 m^2^. Cut-off values for inflammatory parameters predicting in-hospital death derived from ROC analysis are included in Table 3.

Lung tissue consolidations were found in 172 patients (75.1% in whom lung imaging was performed upon arrival to the hospital (230 patients)). Among them, 134 patients (55.8%) had multifocal abnormalities and 59 (24.6%) had a pleural effusion. Ground-glass opacification on CT was present in 38 patients (15.8%).

### 3.2. In-Hospital Analysis

The mean duration of hospital stay was 21.1 ± 12.5 days (median 18; range 3–78). Ninety-five subjects (29.5%) died during hospitalization, with 62 expiring within the first 15 days (65.2% of those who died). The duration of stay varied between in-hospital non-survivors and discharged patients (14.6 ± 11.4 days (median: 11; range: 3–78) vs. 23.9 ± 12.0 days (median: 21; range: 5–69), (*p* < 0.001)). No statistically significant difference was observed between survivors and non-survivors depending on their place of residence.

Comparison of clinical parameters and laboratory results on admission between non-survivors and survivors are presented in Table 1 and Table 2. On average, the in-hospital non-survivors were significantly older than discharged patients (*p* < 0.01). In addition, patients with cognitive impairment and poor functional capacity belonged more frequently to this group (both, *p* < 0.001).

There were also differences in comorbidities. For example, the frequency of heart diseases (but not hypertension) was more common among in-hospital non-survivors; similarly, diabetes (*p* <0.01), cerebral vascular disease (*p* <0.01), and renal disease (*p* <0.01) were more frequent.

In comparison to discharged patients, in-hospital non-survivors had lower hemoglobin levels, with more patients exhibiting low hemoglobin (levels *n* = 71 (75.5%) vs. *n* = 109 (49.6%) (*p* < 0.001)). On admission, inflammatory parameters were also higher among non-survivors, with a number of patients having increased values for selected parameters (neutrophils: *n* = 13 (22.4%) vs. *n* = 18 (10.7%), *p* < 0.05; CRP: *n* = 92 (99.7%) vs. *n* = 183 (83.9%), *p* < 0.001; PCT: 89 (98.9%) vs. *n* = 167 (86.5%), *p* < 0.001; and IL-6: *n* = 80 (94.1%) vs. *n* = 157 (77.0%), *p* < 0.001). Additionally, the frequency of renal impairment, defined as an eGFR below 60 mL/min/1.73 m^2^, was also higher in this group (*n* = 40 (43.0%) vs. *n* = 66 (29.9%), *p* < 0.05).

### 3.3. Post-Discharge Period Analysis

During the post-discharge period (defined as up to 180 days from hospital admission following discharge), an additional 30 patients (9.3%) expired. Among them, 14 died within the first 30 days and the next 6 from 31 to 60 days. Overall, the survivors’ group consisted of 197 patients (61.2%).

Patients who expired after discharge more often had cognitive impairment (*p* < 0.05) and poor functional capacity before contracting COVID-19 (*p* < 0.05 and *p* < 0.001, respectively) as compared to those who survived the analysis period. These were the only differences found between those two groups.

### 3.4. Factors Affecting Mortality

The analysis of factors affecting mortality is presented in Table 4.

Only the parameters which differed between non-survivors and survivors were included in the analysis of survival. In the multivariate Cox proportional hazard analyses, increased risk of in-hospital mortality was associated with severe cognitive impairment on admission, heart disease morbidity, and increased frequency of abnormalities in selected laboratory parameters, such as low hemoglobin and high IL-6. Mortality was approximately 2% higher in males.

**Figure 1:**
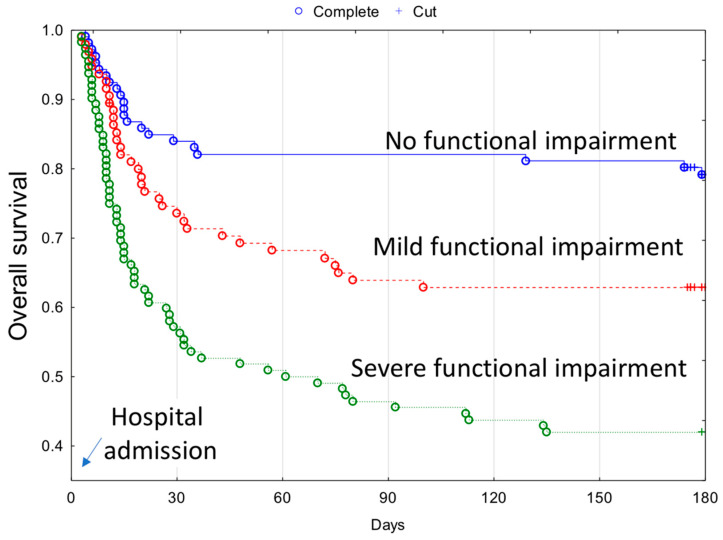
Kaplan–Meier curves depicting the overall 180-day survival rate in COVID-19 patients with coexisting functional impairment prior to contracting COVID-19.

The overall 180-days mortality was associated with similar risk factors as in-hospital mortality, except gender. Additionally, the overall 180-days mortality was higher in patients with poor functional capacity prior to COVID-19 diagnosis. Kaplan–Meier curves of overall 180-day survival across functional capacity are presented in Figure 1.

When the post-discharge period was analyzed separately, poor functional capacity was found as the only factor influencing mortality. Poor functional capacity was more than seven times higher in those with severe disability and almost three times as much in those with mild disability.

## 4. Discussion

This report presents mortality risk factors observed among older individuals who were hospitalized for COVID-19 within three time periods: during hospitalization, 180 days from hospital admission, and separately only after discharge. Our analysis includes 322 consecutive older patients admitted from the beginning of the pandemic up to the end of June 2020 in one of several COVID-19-dedicated hospitals in Poland.

In our study, male sex, severe cognitive impairment, underlying heart disease, anemia, and high plasma levels of IL-6 were independently associated with greater mortality during hospitalization in older patients infected with COVID-19. During the 180-day observational period, similar characteristics, except for male sex and additionally functional impairment, were associated with increased mortality. At post-discharge, severe functional impairment remained as the only determinant.

The observed impact of gender on survival following COVID-19 is in line with a meta-analysis by Peckham et al., which revealed that men were at a higher risk of death [16]. However, in our analysis, this parameter was associated with borderline statistical significance and during hospitalization only.

In our study, severe cognitive impairment increased mortality risk more than four times during hospitalization and more than three times during the period of 180 days from admission. These figures are consistent with a meta-analysis which showed that dementia enhanced the severity and mortality risk of COVID-19 [17]. Moreover, both Bianchetti et al. and Covino et al. reported that dementia was an essential risk factor of mortality for older patients with COVID-19 [18,19].

Research has established comorbidities as crucial risk factors for COVID-19 progression [20,21]. However, not all comorbid conditions in our study revealed robust predictive properties. Our results show that only patients with heart disease had significantly higher mortality during hospitalization and the overall observation period. These findings are in line with a study by España et al., who demonstrated that patients with underlying cardiovascular disease were the most likely to die among a cohort of patients affected by any comorbidities and COVID-19 [22]. Numerous studies have found that patients with underlying heart disease are at increased risk of a more severe clinical course of SARS-CoV-2 infection in addition to higher mortality rates [23,24]. Contributory mechanisms include a systemic inflammatory cytokine storm that mediates atherosclerosis, which in turn destabilizes coronary plaques, induces procoagulant factors, reduces coronary blood flow, and aggravates hypoxia [25,26,27]. Additionally, the virus invades host cells through the angiotensin-converting enzyme 2 (ACE-2) receptors expressed within the cardiovascular and respiratory systems. Subsequently, the virus may directly cause damage to the myocardium and lungs, which in turn increases the severity of infection and risk of death [28].

Another crucial independent mortality factor observed during hospitalization and throughout the overall observational period was anemia. Tao et al. found that anemia was an independent risk factor linked with the severity of COVID-19, while Faghih Dinevari et al. and Tao et al. reported that anemia was independently associated with greater mortality [29,30].

While abnormal laboratory markers were commonly seen in non-survivors, the most important proved to be increased levels of IL-6, as this parameter was independently associated with higher mortality. Elevated IL-6 levels correlated with worsening clinical outcomes in older patients during hospitalization, as well as the overall 180-day observational period. A meta-analysis by Coomes et al. found that patients with complicated cases of COVID-19 had threefold higher IL-6 plasma levels than patients with noncomplicated disease. These levels were also associated with an unfavorable clinical outcome [31]. SARS-CoV-2 has been observed to trigger an aggressive dysregulated host immune response. IL-6 is likely a crucial mediator of respiratory failure, shock, and multiorgan dysfunction [31,32].

Assessment of frailty has been found to be useful in prognostication in older adults affected by COVID-19 [33,34]. Although level of frailty assessed in the elderly prior to COVID-19 infection correlated with the severity of clinical consequences, scores acquired from various frailty assessment scales were not consistent in accordance with certain outcome variables [35]. Limitations regarding the predictive value of frailty evaluation in COVID-19 patients were emphasized by Moug et al. and included subjectivity as well as the restricted specificity of this method [36]. Moreover, a study from Belgium showed that frailty and age alone provide poor specificity for prediction of mortality in older individuals affected by SARS-CoV-2 infection [37]. Therefore, we decided not to include frailty as a parameter within this study.

A key aspect of this work is that patients were followed not only during hospitalization but also in the post-discharge period for up to 180 days from admission. Few studies have included such long observational periods [38,39]. Another feature within this report was that functional capacity of older patients, as reported prior to hospitalization, was documented. This prehospitalization assessment was used as the baseline for functional capacity rather than cognition at the time of admission. Patients were admitted at various stages of disease. We thus decided to assess their independence in the period before contracting the disease. We found functional capacity before hospitalization to be the only independent factor that increases mortality in long-term follow-up by as much as seven times.

Impaired functional status prior to admission was not linked to a poor in-hospital prognosis amongst our cohort. Therefore, our study does not suggest the use of functional status prior to contracting COVID-19 in short-term prognostication. The impact of functional status on in-hospital survival of older adults affected by COVID-19 has already been documented in the literature. Previous studies have found that an inability to perform basic activities of daily living (ADL) on admission increases in-hospital mortality and suggests that assessment of ADLs should be a factor in the decision-making process and management of patients infected with COVID-19 [40,41,42,43]. To the best of our knowledge, this is the first report highlighting the need to consider preadmission functional capacity when evaluating older patients infected with COVID-19 for long-term prognoses. We observed this same phenomenon previously, in a smaller group of patients, with a shorter (60-day) follow-up period [12]. Preadmission functional capacity can, in turn, be used by providers as one indicator of the need for more stringent follow-up upon discharge. Patients with functional impairments who are discharged should be scheduled for sooner and more frequent phone consultations as well as outpatient visits and assessments. At home, caregivers should also be counseled to recognize signs and symptoms of worsening disease in these patients and for the need to promptly seek medical care in certain situations.

The main limitation of our study is that functional status was not assessed using a validated tool. However, our classification was based on ADL and IADL scoring systems. Furthermore, functional status assessment performed by EAMA members has already been used in our previous analysis [12]. Because the aforementioned assessment was conducted retrospectively based on an admission medical history, precise evaluation with scoring was not possible. Moreover, an extensive functional status assessment could have been compromised by patients’ neurocognitive disturbances [44]. In addition, it should be mentioned that attending physicians who admitted patients were not geriatric professionals. For the same reason, cognitive disturbances were not assessed with the use of a validated tool either. Additionally, the distinction between delirium and dementia in the clinical setting is difficult to discern, especially during emergency admissions [45]. Consequently, delirium and early/middle stage dementia were classified together as mild cognitive disturbances. The investigation involved a single center, and therefore, our results cannot be directly generalized. The sample was also largely homogenous (white Caucasians), and the results may not be fully reflective of the presentation of disease in different populations. Finally, given the goal of the study, mortality was the only measured clinical outcome. Therefore, secondary outcomes, such as respiratory insufficiency, need for mechanical ventilation or non-invasive ventilation, and multiorgan failure, were not considered in the analysis.

## 5. Conclusions

Patients with severe functional impairment before contracting COVID-19 were more likely to die following discharge and up to 180 days following hospital admission. Therefore, functional capacity prior to diagnosis should be taken into consideration when formulating comprehensive prognoses for older people with COVID-19. Patients with impairments should be prescribed more stringent and comprehensive follow-up care, as compared to their counterparts who present without these symptoms at the time of diagnosis of COVID-19.

## Figures and Tables

**Table 1:** Characteristics of analyzed patients on admission (*n* = 322) ^1^.

Characteristic	Total	In-Hospital ^2^			Post-Discharge ^3^		
		Non-Survivors	Survivors	*p*-Value	Non-Survivors	Survivors	*p*-Value
Age	77.5 ± 10.0 (77; 60–101)	79.7 ± 8.9 (79; 63–99)	76.6 ± 10.3 (75; 60–101)	*p* < 0.01	80.2 ± 11.1 (79.5; 61–101)	76.0 ± 10.1 (74; 60–99)	0.06
Gender							
Male	131 (40.7%)	46 (48.4%)	85 (37.4%)	0.07	9 (30.0%)	76 (38.6%)	0.37
Female	191 (59.3%)	49 (51.6%)	142 (62.6%)		21 (70.0%)	121 (61.4%)	
Symptoms							
● Subfebrile Temperature	94 (32.8%)	26 (33.8%)	68 (33.4%)	0.82	7 (25.0%)	60 (33.5%)	0.39
● Fever	33 (11.5%)	10 (13.0%)	23 (11.1%)	0.63	0 (0.0%)	23 (12.6%)	0.95
● Cough	66 (26.4%)	12 (18.5%)	54 (29.2%)	0.09	3 (13.6%)	51 (31.3%)	0.14
● Dyspnea	54 (20.5%)	18 (25.0%)	36 (18.8%)	0.26	3 (12.5%)	33 (19.6%)	0.58
● Dysosmia	2 (1.3%)	1 (2.9%)	1 (0.8%)	0.34	0 (0.0%)	1 (1.0%)	0.25
● Dysgeusia	5 (3.3%)	1 (2.9%)	4 (3.3%)	0.91	0 (0.0%)	4 (3.8%)	1.00
● Myalgia	15 (6.3%)	5 (7.8%)	10 (5.8%)	0.79	0 (0.0%)	10 (6.6%)	0.45
Cognitive function							
● Cognitively intact	172 (53.9%)	33 (34.7%)	139 (62.1%)	*p* < 0.0001	12 (41.4%)	127 (65.1%)	<0.05
● Mild impairment	117 (36.7%)	42 (44.2%)	75 (33.5%)		13 (44.8%)	62 (31.8%)	
● Severe impairment	30 (9.4%)	20 (21.1%)	10 (4.5%)		4 (13.8%)	6 (3.1%)	
Functional capacity ^1^							
● No functional impairment	110 (34.5%)	18 (19.0%)	92 (41.1%)	*p* < 0.0001	4 (13.8%)	88 (45.1%)	<0.001
● Mild functional impairment	97 (30.4%)	29 (30.5%)	68 (30.3%)		8 (27.6%)	60 (30.8%)	
● Severe functional impairment	112 (35.1%)	48 (50.5%)	64 (28.6%)		17 (58.6%)	47 (24.1%)	
Comorbidities							
Cardiovascular diseases	265 (82.3%)	84 (88.4%)	181 (79.7%)	0.63	23 (76.7%)	158 (80.2%)	0.65
● Hypertension	199 (62.1%)	57 (60.0%)	142 (63.4%)	0.57	15 (50.0%)	127 (65.5%)	0.10
● Heart diseases	189 (59.3%)	67 (70.5%)	122 (54.5%)	<0.01	20 (66.7%)	102 (52.6%)	0.15
Diabetes	99 (31.0%)	40 (42.1%)	59 (26.3%)	<0.01	8 (26.7%)	51 (26.3%)	0.97
Respiratory diseases	30 (9.4%)	13 (13.7%)	17 (7.6%)	0.13	5 (16.7%)	12 (6.2%)	0.10
Renal dysfunction	49 (15.4%)	22 (23.2%)	27 (12.1%)	<0.05	5 (16.7%)	22 (11.3%)	0.59
Liver dysfunction	10 (3.1%)	4 (4.2%)	6 (2.7%)	0.71	2 (6.7%)	4 (2.1%)	0.40
Cancer	30 (9.4%)	11 (11.6%)	19 (8.5%)	0.39	5 (16.7%)	14 (7.2%)	0.17
Stroke	63 (19.8%)	27 (28.4%)	36 (16.1%)	<0.05	7 (23.3%)	29 (15.0%)	0.32

^1^ Functional capacity was assessed based on the data prior to contracting COVID-19. ^2^ In-hospital columns characterize the patients on admission with the division into those who died during hospitalizations and those who were discharged. ^3^ Thereafter, discharged patients were divided into those who died after discharge and those who survived by the end of the observation period; this analysis is presented in the last 3 columns.

**Table 2:** Laboratory parameters on admission (*n* = 322).

Parameter	Total	In-Hospital ^1^			Post-Discharge ^2^		
		Non-Survivors	Survivors	*p*-Value	Non-Survivors	Survivors	*p*-Value
White Blood Cells (×10^3^/µL) 4.0–11.0	7.3 ± 3.5 (6.8; 1.7–29.0)	8.4 ± 4.6 (7.5; 1.7–29.0)	6.8 ± 2.9 (6.5; 1.7–17.1)	*p* < 0.01	7.8 ± 3.3 (7.6; 3.2–17.1)	6.7 ± 2.8 (6.2; 1.7–14.8)	0.07
Hemoglobin (g/dL) Female 12–16	11.9 ± 1.7 (12.1; 6.7–15.3)	11.4 ± 2.0 (11.3; 7.3–15.2)	12.0 ± 1.6 (12.3; 6.7–15.3)	*p* < 0.05	11.6 ± 2.0 (12.7; 8.1–14.0)	12.1 ± 1.5 (12.2; 6.7–15.3)	0.54
Hemoglobin (g/dL) Male 14–18	12.5 ± 2.0 (12.7; 6.4–17.3)	11.5 ± 2.1 (11.7; 6.5–15.4)	13.0 ± 1.8 (13.1; 6.4–17.3)	*p* < 0.001	11.6 ± 2.6 (12.1; 6.4–15.6)	13.1 ± 1.7 (13.2; 9.1–17.3)	*p* < 0.05
Platelets (×10^3/^µL) 130–440	243.3 ± 109.2 (221.5; 35.0–867.0)	236.2 ± 124.6 (204.5; 35.0–867.0)	246.4 ± 102.1 (227.5; 61.0–665.0)	0.18	257.2 ± 91.9 (263.0; 85.0–465.0)	244.7 ± 103.7 (226.0; 61.0–665.0)	0.28
Lymphocytes (×10^3^/µL) 1.0–4.0	1.4 ± 0.8 (1.2; 0.0–8.1)	1.3 ± 1.1 (1.1; 0.3–8.1)	1.4 ± 0.7 (1.2; 0.0–3.8)	0.07	1.4 ± 0.7 (1.2; 0.5–2.6)	1.4 ± 0.7 (1.2; 0–3.8)	0.74
Neutrophils (×10^3^/µL) 1.5–7.7	4.9 ± 2.9 (4.3; 0.1–16.9)	6.0 ± 3.7 (5.1; 0.1–16.9)	4.6 ± 2.4 (4.1; 0.7–12.9)	*p* < 0.01	4.9 ± 2.3 (4.1; 2.4–11.1)	4.5 ± 2.5 (4.0; 0.7–12.9)	0.40
Urea (mmol/L) Female 3.6–7.1	8.1 ± 6.9 (6.1; 2.0–45.2)	10.6 ± 8.0 (9.0; 2.0–31.8)	7.3 ± 6.4 (5.8; 2.5–45.2)	*p* < 0.05	10.1 ± 11.4 (6.8; 3.1–45.2)	6.9 ± 5.1 (5.7; 2.5–37.9)	0.17
Urea (mmol/L) Male 3.2–8.9	9.6 ± 9.5 (6.9; 1.4–69.1)	13.4 ± 13.8 (9.7; 3.8–69.1)	7.3 ± 3.7 (6.3; 1.4–20.4)	*p* < 0.01	10.9 ± 5.6 (9.7; 5.1–20.4)	6.8 ± 3.1 (6.1; 1.4–15.7)	*p* < 0.05
Lactate Dehydrogenase (U/L) 125–220	326.5 ± 159.8 (289.5; 5.3–1332.0)	388.8 ± 215.3 (346.0; 5.3–1332.0)	300.9 ± 122.0 (279.0; 133.0–856.0)	*p* < 0.001	288.6 ± 126.8 (262.0; 138.0–762.0)	302.8 ± 121.6 (283.0; 133.0–856.0)	0.46
CRP (mg/L) 0–5	70.9 ± 76.3 (44.9; 0.0–455.0)	102.3 ± 92.8 (71.8; 2.4–455.0)	57.4 ± 63.5 (34.3; 0.0–290.5)	*p* < 0.001	54.2 ± 54.5 (36.0; 0.0–176.1)	57.9 ± 64.9 (34.0; 0.0–290.5)	0.76
PCT (ng/mL) 0–0.1	0.7 ± 4.4 (0.1; 0.0–49.1)	1.7 ± 7.1 (0.1; 0.0–49.1)	0.3 ± 2.2 (0.0; 0.0–30.3)	*p* < 0.001	0.4 ± 0.8 (0.0; 0.0–4.1)	0.3 ± 2.4 (0.0; 0.0–30.3)	0.11
IL-6 (pg/mL) 1.5–7.0	71.5 ± 154.0 (28.4; 0.0–1592.0)	147.3 ± 253.4 (60.6; 0.0–1592.0)	39.9 ± 60.5 (18.3; 0.0–500.0)	*p* < 0.001	40.0 ± 36.3 (24.2; 0.0–127.1)	39.9 ± 63.7 (17.7; 0.0–500.0)	0.21

^1^ In-hospital columns characterize the patients on admission with the division between those who died during hospitalizations and those who were discharged. ^2^ Thereafter, discharged patients were divided between those who died after discharge and those who survived by the end of the observational period; this analysis is presented in the last 3 columns.

**Table 3:** Cut-off values for inflammatory parameters predicting in-hospital death based on ROC analysis. No cut-off value was showed for PCT in this analysis.

Parameter	Cut-Off	Sensitivity (%)	Specificity (%)
WBC (×10^3^/µL)	>10.0	33.3	100.0
Neutrophils (×10^3^/µL)	>5.0	66.7	80.0
CRP (mg/L)	>108.0	55.6	89.2
Il-6 (pg/mL)	>29.0	72.7	82.1

**Table 4:** Factors affecting in-hospital, post-discharge, and overall 180-day mortality in Cox proportional hazard analyses. Data are shown as hazard ratios (HR) with 95% confidence intervals.

Factor	Feature	In-Hospital Mortality		Post-Discharge Mortality		Overall 180-Day Mortality	
		Univariable Model	Multivariable Model	Univariable Model	Multivariable Model	Univariable Model	Multivariable Model
Age	≥75	1.64 (1.07–2.51); *p* < 0.05	-	1.51 (0.72–3.17); *p* = 0.27	-	1.65 (1.14–2.38); *p* < 0.01	-
Sex	M	1.02 (1.00–1.04); *p* < 0.05	1.02 (1.00–1.04); *p* < 0.05	0.62 (0.28–1.41); *p* = 0.26	-	1.07 (0.39–2.96); *p* = 0.89	-
Functional impairment	Mild	1.46 (0.80–2.66); *p* = 0.22	-	2.90 (0.87–9.65); *p* = 0.08	2.90 (0.87–9.65); *p* = 0.08	2.02 (1.19–3.45); *p* < 0.01	1.96 (1.10–3.48); *p* < 0.05
	Severe	2.49 (1.44–4.29); *p* < 0.01	-	7.02 (2.36–20.90); *p* < 0.001	7.02 (2.36–20.90); *p* < 0.001	3.80 (3.34–6.17); *p* < 0.001	2.92 (1.68–5.06); *p* < 0.001
Cognitive impairment	Mild	1.73 (1.08–2.76); *p* < 0.05	-	2.20 (1.00–4.84); *p* < 0.05	-	2.05 (1.38–3.06); *p* < 0.001	-
	Severe	4.29 (2.45–7.49); *p* < 0.001	4.42 (2.59–7.53); *p* < 0.001	5.19 (1.67–16.11); *p* < 0.01	-	5.10 (3.09–8.43); *p* < 0.001	3.10 (1.84–5.25); *p* < 0.001
Diabetes	-	1.55 (1.02–2.34); *p* < 0.05	-	1.10 (0.49–2.48); *p* = 0.82	-	1.57 (1.09–2.25); *p* < 0.05	-
Heart disease	-	1.86 (1.19–2.92); *p* < 0.01	2.17 (1.34–3.54); *p* < 0.01	1.68 (0.78–3.60); *p* = 0.19	-	1.78 (1.22–2.61); *p* < 0.01	1.99 (1.32–2.99); *p* < 0.01
History of kidney disease	-	1.37 (0.84–2.27); *p* = 0.21	-	1.57 (0.60–4.13); *p* = 0.36	-	1.66 (1.08–2.57); *p* < 0.05	-
eGFR	<60	1.86 (1.22–2.82); *p* < 0.01	-	0.86 (0.38–1.94); *p* = 0.71	-	1.46 (1.01–2.09); *p* < 0.05	-
Past stroke	-	1.50 (0.96–3.33); *p* = 0.08	-	1.64 (0.70–3.86); *p* = 0.25	-	1.75 (1.19–2.58); *p* < 0.01	-
Anemia	-	2.48 (1.55–3.99); *p* < 0.001	2.21 (1.33–3.68); *p* < 0.01	1.20 (0.57–2.52); *p* = 0.63	-	2.17 (1.47–3.20); *p* < 0.001	1.97 (1.30–2.99); *p* < 0.01
WBC	>10.0	2.51 (1.48–4.29); *p* < 0.001	-	1.66 (0.58–4.80); *p* = 0.35	-	1.96 (1.22–3.13); *p* < 0.01	-
Neutrophiles	>5.0	1.99 (1.18–3.36); *p* = 0.01	-	1.09 (0.41–2.89); *p* = 0.87	-	1.62 (1.03–2.55); *p* < 0.05	-
CRP	>100	1.86 (1.21–2.85); *p* < 0.01	-	1.11 (0.47–2.61); *p* = 0.81	-	1.52 (1.04–2.22); *p* < 0.05	-
IL6	≥30	3.81 (2.34–6.22); *p* < 0.001	3.49 (2.13–5.74); *p* < 0.001	1.65 (0.79–3.47); *p* = 0.19	-	3.09 (2.08–4.58); *p* < 0.001	3.18 (2.12–4.77); *p* < 0.01

## Data Availability

The data presented in this study are available on request from the corresponding author. The data are not publicly available due to General Data Protection Regulation (EU GDPR).
